# Supplementary material for: Mental Health Disparities among Pre-Clinical Medical Students at Saint Louis University during the COVID-19 Pandemic
Source: Behav Sci (Basel). 2024 Jan 26;14(2):89. doi: 10.3390/bs14020089 (PMC10885875; doi:10.3390/bs14020089)
Supplement: Supplementary file 1 [file behavsci-14-00089-s001.zip › behavsci-2742969-supplementary.pdf]

Table S1. Mental health characteristics among those at-risk for anxiety and depression, stratified by severity. Severe depression and anxiety were defined by a PHQ-9 score of 20 or greater, and a GAD-7 score of 15 or greater, respectively. URM = underrepresented in medicine. PHQ-9: Patient Health Questionnaire-9. GAD-7: Generalized Anxiety Disorder-7.

	URM (n=7)	Non URM (n=29)
Anxiety Severity		
Severe	2 (28.6%)	5 (17.2%)
Non-severe	5 (71.4%)	24 (82.8%)
Depression Severity		
Severe	1 (14.3%)	3 (10.3%)
Non-severe	6 (85.7%)	26 (89.7%)

Table S2. A. Multiple regression table of PHQ-9 scores with institutional concerns as predictors. B. Multiple regression table of GAD-7 scores with institutional concerns as predictors. PHQ-9: Patient Health Questionnaire-9. GAD-7: Generalized Anxiety Disorder-7. ANOVA = analysis of variance. B = unstandardized Beta coefficient. df = degrees of freedom. Sig. = significance.

A. PHQ-9

ANOVA

**Model**	**Sum of squares**	**df**	**Mean square**	**F**	**Sig.**
Regression	727.163	5	145.433	5.965	<0.001
Residual	1974.768	81	24.380		
Total	2701.931	86			

Coefficients

Model	B	Standard error	Beta	t	Sig.
(Constant)	2.725	0.853		3.193	0.002
Concern of lack of academic support	-0.019	0.032	-0.095	-0.606	0.546
Concern of lack of mental health support	0.059	0.031	0.285	1.862	0.066
Concern of lack of financial support	0.044	0.019	0.251	2.362	0.021
Concern of delayed medical training	-0.020	0.035	-0.081	-0.562	0.575
Concern of decreased quality of medical training	0.065	0.030	0.314	2.142	0.035

B. GAD-7

ANOVA

Model	Sum of squares	df	Mean square	F	Sig.
Regression	556.621	5	111.324	4.939	<0.001
Residual	1825.816	81	22.541		
Total	2382.437	86			

Coefficients

Model	B	Standard error	Beta	t	Sig.
(Constant)	3.096	0.821		3.773	<0.001
Concern of lack of academic support	0.020	0.031	0.104	0.647	0.520
Concern of lack of mental health support	0.067	0.030	0.347	2.210	0.030
Concern of lack of financial support	0.003	0.018	0.019	0.176	0.860
Concern of delayed medical training	-0.054	0.034	-0.237	-1.609	0.111
Concern of decreased quality of medical training	0.053	0.029	0.275	1.832	0.071

Table S3. A. Multiple regression table of PHQ-9 scores with pandemic-related concerns as predictors. B. Multiple regression table of GAD-7 scores with pandemic-related concerns as predictors. PHQ-9: Patient Health Questionnaire-9. GAD-7: Generalized Anxiety Disorder-7. ANOVA = analysis of variance. B = unstandardized Beta coefficient. df = degrees of freedom. Sig. = significance.

**A. PHQ-9**					
**ANOVA**					
**Model**	**Sum of squares**	**df**	**Mean square**	**F**	**Sig.**
Regression	671.415	4	167.854	6.779	<0.001
Residual	2030.516	82	24.762		
Total	2701.931	86			
**Coefficients**					
Model	B	Standard error	Beta	t	Sig.
(Constant)	2.539	1.209		2.101	0.039
Concern of lack of social interaction	0.044	0.017	0.255	2.517	0.014
Concern of contracting COVID	0.018	0.023	0.105	0.807	0.422
Concern of transmitting COVID	-0.016	0.022	-0.098	-0.737	0.463
Concern of new financial difficulty	0.084	0.022	0.387	3.889	<0.001

**B. GAD-7**					
**ANOVA**					
Model	Sum of squares	df	Mean square	F	Sig.
Regression	287.016	4	71.754	2.808	0.031
Residual	2095.420	82	25.554		
Total	2382.437	86			
**Coefficients**					
Model	B	Standard error	Beta	t	Sig.
(Constant)	2.795	1.228		2.276	0.025
Concern of lack of social interaction	0.023	0.018	0.141	1.291	0.200
Concern of contracting COVID	0.016	0.023	0.095	0.678	0.499
Concern of transmitting COVID	0.007	0.022	0.043	0.300	0.765
Concern of new financial difficulty	0.046	0.022	0.226	2.097	0.039

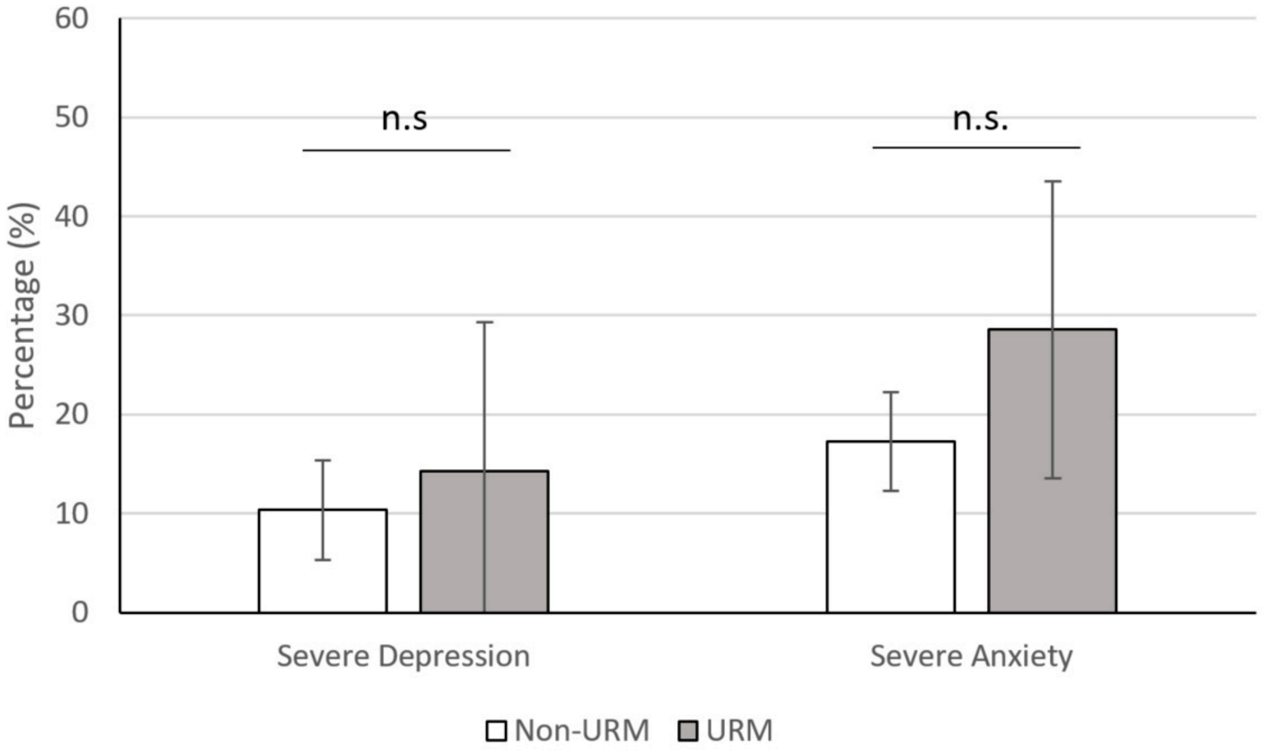

Figure S1. Left: Percentage of survey participants who were at-risk of depression that met criteria for severe depression based on PHQ-9 scores, with a threshold of 20. Right: Percentage of survey participants who were at-risk of anxiety that met criteria for severe anxiety based on GAD-7 scores, with a threshold of 15. Statistical significance was calculated via chi-squared analyses. Error bars indicate standard error. PHQ-9: Patient Health Questionnaire-9. GAD-7: Generalized Anxiety Disorder-7. URM = underrepresented in medicine. n.s. = not significant, p = 0.766 (left), p = 0.497 (right).
